# Morphine administration during low ovarian hormone stage results in transient over expression of fear memories in females

**DOI:** 10.3389/fnbeh.2015.00129

**Published:** 2015-05-22

**Authors:** Emily M. Perez-Torres, Dinah L. Ramos-Ortolaza, Roberto Morales, Edwin Santini, Efrain J. Rios-Ruiz, Annelyn Torres-Reveron

**Affiliations:** ^1^School of Behavioral and Brain Sciences, Ponce Health Sciences University - Ponce Research InstitutePonce, Puerto Rico; ^2^Department of Biology, Pontifical Catholic University of Puerto RicoPonce, Puerto Rico; ^3^Department of Counseling and Psychological Services, Institute of Translational Research in Behavioral Sciences, University of Puerto Rico at PoncePonce, Puerto Rico; ^4^Department of Pharmaceutical Science, Palm Beach Atlantic UniversityWest Palm Beach, FL, USA; ^5^Basic Sciences Division, Physiology, Ponce Health Sciences University - Ponce Research InstitutePonce, Puerto Rico

**Keywords:** fear conditioning, fear extinction, morphine, trauma, sex-differences, estrous cycle

## Abstract

Acute exposure to morphine after a traumatic event reduces trauma related symptoms in humans and conditioned fear expression in male rats. We aimed to determine whether acute administration of morphine alters consolidation of fear learning and extinction. Male and female rats in proestrus and metaestrus (high and low ovarian hormones respectively) underwent fear conditioning and received saline or morphine (2.5 mg/kg s.c.). The next day they underwent extinction. Results showed increased freezing during extinction only in the morphine metaestrus group while morphine did not affect males or proestrus females. Recall of extinction was similar on all groups. On a second experiment, a subset of rats conditioned during metaestrus was administered morphine prior to extinction producing no effects. We then measured mu opioid receptor (MOR) expression in the amygdala and periaqueductal gray (PAG) at the end of extinction (day 2). In males and proestrus females, morphine caused an increase in MOR in the amygdala but no in the PAG. In metaestrus females, morphine did not change MOR expression in either structure. These data suggests that ovarian hormones may interact with MORs in the amygdala to transiently alter memory consolidation. Morphine given after trauma to females with low ovarian hormones might increase the recall of fear responses, making recovery harder.

Studies with female and male rats regarding fear conditioning and extinction have demonstrated that ovarian hormones modulate fear acquisition and fear extinction (Milad et al., 2009). The higher fear observed in female in comparison to male rats during extinction recall (Milad et al., 2009), suggests an important role in the ovarian hormones in the higher prevalence of anxiety disorders in women (McLean et al., 2011). In the Pavlovian fear conditioning paradigm repeated pairings of a neutral stimulus such as a tone (conditioned stimulus, CS) with an aversive stimulus such as a mild foot shock (unconditioned stimulus, US) lead to conditioned fear responses such as freezing. However, repeated presentations of the CS in the absence of the US will lead to a gradual reduction in conditioned fear responses commonly known as extinction. Extinction does not erase the initial CS-US association, but is thought to form a new inhibitory memory (Pavlov, 1928; Konorski, 1967). Deficits in fear extinction are thought to contribute to trauma related disorders (Milad et al., 2006, 2009; Glover et al., 2012).

Opiates like morphine, which preferentially bind to the mu opioid receptor (MOR), are one of the first line prescriptions for severe physical traumas, mostly used to ameliorate pain. Despite the large usage of morphine, little is known about the association of mental health disorders and its prescription (Seal et al., 2012). There are several reasons to believe that pharmacotherapy plays an important role in the development of trauma related disorders (Bailey et al., 2013). Clinical studies have found that acute administration of morphine has a protective effect in Posttraumatic Stress Disorder (PTSD) patients by preventing symptoms associated to the disorder and the diagnosis (Bryant et al., 2009). Endogenous opioids may be involved in certain symptoms of trauma related disorders such as numbing, stress-induced analgesia, and dissociation (Holbrook et al., 2010). Taking into considerations these clinical studies, it is evident that not only they support a role of the opioid system in trauma related disorders, but also suggest that this system could be a therapeutic target.

The circuitry of fear learning and extinction has been well mapped. The basolateral amygdala (BLA) is involved in extinction learning by stimulating inhibitory intercalated cell mass activity to inhibit the central amygdala output neurons (McNally and Westbrook, 2003; McNally et al., 2004; Likhtik et al., 2008; Parsons et al., 2010). This intercalated cells are rich in MORs (Likhtik et al., 2008). In addition, there is evidence in humans and animal suggests that estrogens may exert their influence on fear within the amygdala (Jasnow et al., 2006). Estradiol also stimulates the release of endogenous opioid peptides in the medial amygdala (Eckersell et al., 1998). Besides the amygdala, the periaqueductal gray (PAG) matter has an important role in the expression of freezing behavior (Amorapanth et al., 1999). The PAG is rich in opioid receptors (McNally, 2009) and shows sexual dimorphism (Loyd and Murphy, 2006). Opioids within the ventrolateral periaqueductal gray (vlPAG) are necessary for extinction acquisition and blocking MORs in this region prevented acquisition of extinction (McNally et al., 2005). Opioidergic signaling in the vlPAG affects plasticity across the brain circuit responsible for the formation of extinction memory (Parsons et al., 2010).

The aim of this study is to elucidate if acute morphine administration will have a stronger effect in reducing fear conditioning in female rats as compared to male rats. We also aim to know if the behavioral differences will depend on changes in MOR expression in the amygdala and the PAG in response to an opioid agonist.

We used female and male Sprague–Dawley rats (230–300 g in weight) were paired housed under a day-night (12-h) cycle. The rats received free access to food and water throughout the experiment and during at least a one-week acclimation period prior to experimentation, where the rats were handle when performing daily vaginal smears to determine the estrous cycle stage in female rats (Turner and Bagnara, 1971). Only rats with regular, 4-day estrous cycles were included in the study. All procedures were conducted in accordance with and approved by the Ponce Health Sciences University Institutional Animal Care and Use Committee.

Experiments 1, and 2 used four identical conditioning chambers (25 cm × 29 cm × 28 cm, l × w × h; Coulbourn Instruments) located inside of a sound-attenuating box (Med-Associates). The chamber floor consists of 0.5 cm stainless steel bars through which an electric shock is delivered. The chamber is equipped with a speaker and a single overhead light. The auditory tone was a 4 kHz sine wave with duration of 30 sand an intensity of 80 dB sound pressure level (Santini et al., 2004). We assessed the animal in the same conditioning chamber, so the fear expressed will not be specific to the tone, but a combination of fear to the tone and to the context. Males and females were assessed in the same chambers; all boxes were thoroughly cleaned with 70% ethanol between animals. All testing sessions occurred between 9:00 AM and 1:00 PM. The three stages of training were as follows, with the habituation and conditioning performed on the same day: habituation; the animals received one habituation trial (tone alone) in the conditioning chamber with an average intertrial interval (ITI) of 2 min and conditioning; the animals received 3 tone-footshock pairings (0.5 s 0.45 mA) in the box/context and returned to their home cages. A group of female rats was in the proestrus stage of the estrous cycle while another group was in the metaestrus stage during conditioning. Twenty-four hours after conditioning, the animals received extinction and consisted of 12 tone alone presentations. Twenty-four hours after the conditioning a sub group of metaestrus females receive a test that consisted of 2 tone alone presentations. Animals in each experiment group were treated with morphine dissolved in saline (2.5 mg/kg) or saline (0.9%) subcutaneously immediately after conditioning or 4 h before extinction. The morphine dose was chosen as it has been shown to be effective in reducing conditioned fear (Rudy et al., 1999). This dose of morphine administered acutely does not provoke withdrawal symptoms. Studies have demonstrated that there are sex differences in rats in response to morphine antinociceptive activity and differences between men and women analgesia (Cicero et al., 1997; Sarton et al., 2000). However, today is not clear the sex-differences in the pharmacokinetics of morphine. Immediately after experiments rats were anesthetized and decapitated, brains removed and frozen. The amygdala and PAG were dissected based on the atlas of Paxinos and Watson. Equal amounts of protein (100 μg) were used to identify the MORs with a rabbit polyclonal antibody (1:1000, Immunostar). Blots were washed with TBST and incubated in goat anti-rabbit IgG (1:1000, Santa Cruz). Bands were visualized using Chemidoc XRS Imaging System and Image J software (imagej.nih.gov). To perform this, we followed Ramos-Ortolaza et al. (2010) western blot protocol. All samples were run in duplicates with saline and morphine groups within the same gel. Our goal was to look at changes caused by morphine and not absolute changes in protein, therefore data was analyzed as a percent change in morphine-treated samples against control group within a given gel. For a subgroup of gels we calculated GAPDH loading control and we found a 2.3% of variability between wells, which equally affected controls and morphine treated animals.

Freezing time per trial was averaged in blocks of two and converted to percentage. We used repeated measures ANOVA considering treatment (morphine or saline), sex (female or male) and female cycle stage (metaestrus or proestrus) as between-subject variables. For all experiments, the significance level was set at *p* < 0.05. Significant interactions were examined using Tukey’s *post hoc* comparisons. The western blot was analyzed using one sample *t*-test against baseline (100%) for differences against saline control group for each sex group. Differences between sexes for morphine groups were analyzed using ANOVA.

Behavioral results show that for the experiment 1, and 2 when morphine (2.5 mg/kg) or saline (0.9%) where administered subcutaneously immediately after fear conditioning (Figures 1A–D) or 4 h prior to extinction (Figures 1E,F) there were no statistical differences in the levels of conditioned freezing between groups designated to receive saline or morphine; metaestrus (*F*_(1,27)_ = 0.412, *p* > 0.05), proestrus (*F*_(1,23)_ = 0.009, *p* > 0.05) and males (*F*_(1,28)_ = 2.12, *p* > 0.05; Figure 1). However, extinction results showed that administration of acute morphine immediately after conditioning caused an increased level of conditioned freezing in the group conditioned during metaestrus compared to controls (*F*_(1,27)_ = 25.41, *p* < 0.01; Figure 1B). No significant differences were observed in the extinction session for males (*F*_(1,28)_ = 1.35, *p* > 0.05) or in female rats conditioned during proestrus (*F*_(1,23)_ = 0.74, *p* > 0.05; Figures 1C,D). To further examine our finding in the metaestrus conditioned group, we selected a separate set of animals that was tested for freezing behavior 24 h after fear extinction (Day 3). This test consisted of two tone-alone presentations. No effects were observed on day 3 for female rats conditioned during metaestrus (*F*_(1,11)_ = 0.63, *p* > 0.05) (not shown), thus they remembered the extinction from previous day. We further analyzed whether the deficit in extinction could be associated with the stage of the cycle alone during extinction (day 2). Most rats (82%) injected with morphine and conditioned during proestrus switched to estrus/metaestrus stages. On the other hand, 79% the animals that were conditioned in metaestrus and treated with morphine stayed in metaestrus and/or switched to diestrus II. Thus, when morphine and saline groups were re-analyzed considering the stage of the cycle at the extinction phase, all rats that receive morphine and were in metaestrus/diestrus II still showed increased fear on Day 2 compared to saline controls. On experiment 2, additional group of metaestrus female rats were given morphine (2.5 mg/kg s.c.) or saline (0.9%) 4 h before extinction (Figure 1E). They were then subjected to a two tone-alone test on Day 3. There were no differences in freezing behavior between groups that received saline or morphine; conditioning (*F*_(1,12)_ = 0.011, *p* > 0.05), extinction (*F*_(1,12)_ = 0.47, *p* > 0.05; Figure 1F) and test (*F*_(1,12)_ = 0.36, *p* < 0.05; not shown).

**Figure 1:**
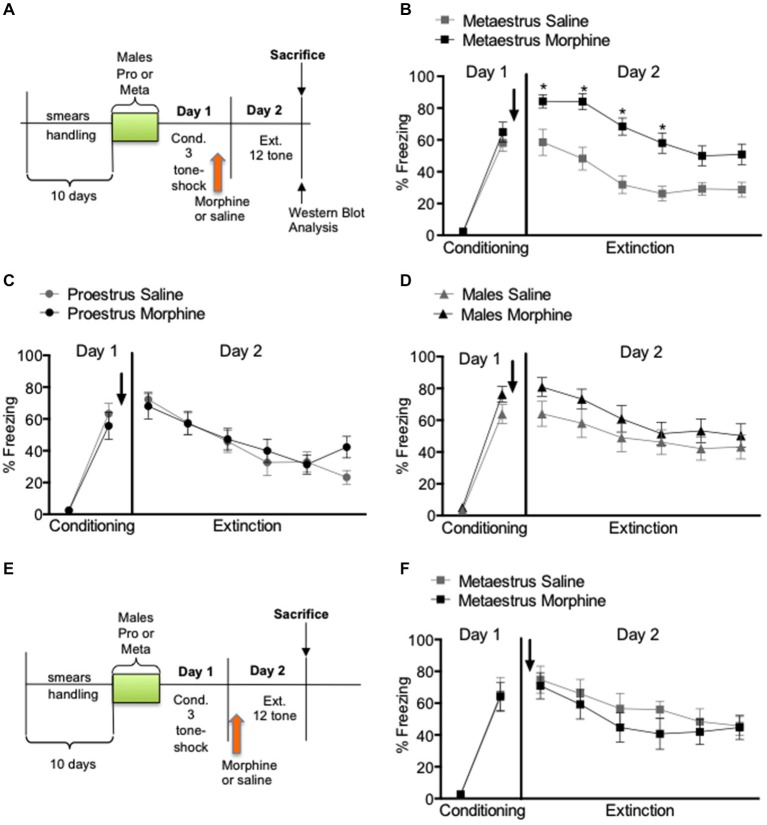
**Percent freezing to the tone in males, proestrus and metaestrus female rats shown in blocks of two trials**. Acute morphine immediately after conditioning resulted in an increased level of conditioned freezing in the metaestrus group compare to controls and acute morphine 24 h after conditioning had no significant effect in extinction learning in metaestrus female rats. **(A)** Experimental timeline. **(B)** Post-training injections of morphine given after the metaestrus stage had no effect on extinction, but over expression of conditioned fear (morphine *n* = 14, saline = 14). **(C)** Post-training injections of morphine given during the proestrus stage had no effect either in the consolidation of fear conditioning or within session extinction (morphine *n* = 13, saline *n* = 14). **(D)** Post-training injections of morphine had no effect either in the consolidation of fear conditioning or within session extinction in male rats (morphine *n* = 15, saline *n* = 14). **(E)** Experimental timeline. **(F)** Pre-extintion injections of morphine given after the metaestrus stage had no effect either in the consolidation of fear conditioning or extinction (morphine *n* = 8, saline *n* = 6).

To quantify how MOR expression was altered in response to morphine, we dissected the amygdala and PAG of the females and male rats from experiment 1, right after the end of the extinction session on Day 2. Western blot results showed that morphine administration to male rats increased MOR expression in the amygdala (*F*_(1,4)_ = 3.323, *p* < 0.05; Figure 2A), but did not affect MOR expression in the PAG (*F*_(1,4)_ = 0.640, *p* > 0.05; Figure 2A). Like in the males, morphine administration increased MOR expression in the amygdala of the proestrus female rats (*F*_(1,4)_ = 5.165, *p* < 0.05) and did not affect MOR expression in the PAG (*F*_(1,4)_ = 0.239, *p* > 0.05; Figure 2B). In contrast, morphine administration to metaestrus female rats did not affect MOR expression in the amygdala (*F*_(1,4)_ = 0.335, *p* > 0.05) or the PAG (*F*_(1,4)_ = 0.065, *p* > 0.05; Figure 2C).

**Figure 2:**
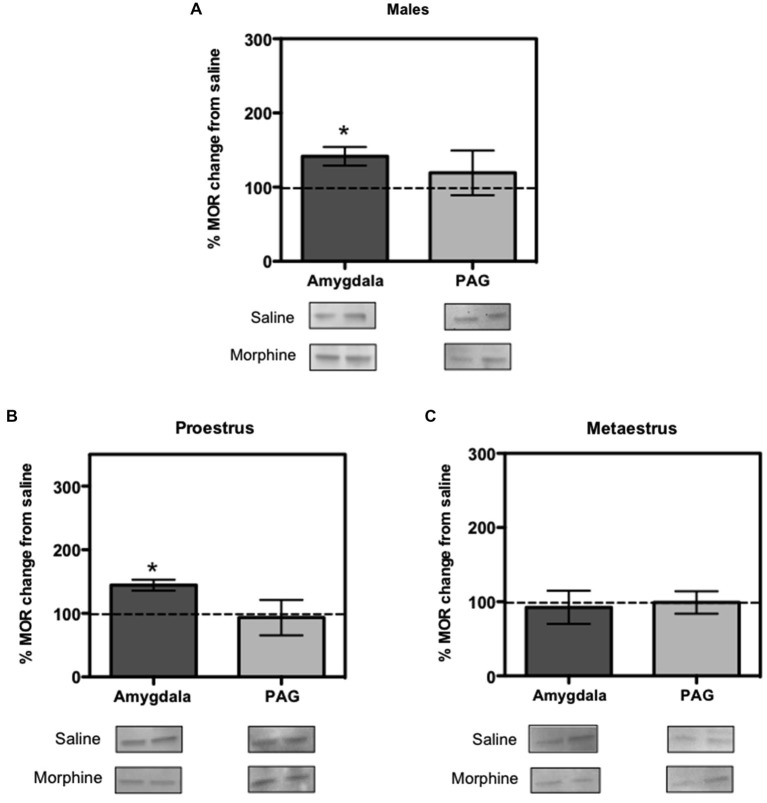
**Ratio of mu opioid receptor (MOR) changes from control group in males, proestrus and metaestrus female rats**. **(A)** In males the amygdala showed an increase in MOR expression produced by morphine (*p* < 0.05 compared to baseline control). **(B)** In proestrus female the amygdala showed an increase in MOR expression produced by morphine (*p* < 0.05). **(C)** In metaestrus female rats the amygdala and periaqueductal gray (PAG) showed no significant difference in MORs expression compared to controls.

The data gathered in this study show a transient over expression of fear memories in female rats fear conditioned and treated with morphine during the metaestrus stage of the estrous cycle. However, there was no significant difference in the recall of extinction memory on metaestrus females suggesting a transient effect of morphine shortly after its administration. Interestingly, when treating the metaestrus rats with morphine before extinction, no effects on fear were observed. This suggests that the effects of acute morphine in females are linked to the events that start shortly after the trauma occurs but once the memory has been consolidated, morphine will have no effect.

Animal studies suggest that gonadal hormones influence extinction of conditioned fear. Milad et al. (2009) showed that female rats during the proestrous stage of the estrus cycle exhibited better extinction memory during extinction recall test. Also, when estradiol and progesterone is administered exogenously there is a facilitated extinction recall, whereas estradiol and progesterone receptor antagonists impair it. This suggests that gonadal hormones influence the consolidation of extinction memory (Milad et al., 2009). It is important to point out, that our saline animals are consistent from Milad et al. (2009) study, since he also analyzed the cycle stage of the female rat during conditioning and did not see differences in behavior between groups.

Studies indicate that low ovarian hormones are associated with higher fear expression (Milad et al., 2009; Glover et al., 2012, 2013). In our study many rats that were in metaestrus during conditioning continued in the same cycle stage or entered in diestrus II during extinction. We found that the cycle alone cannot explain the over expression of fear because it is not reduced when only the rats that transitioned out of the metaestrus/diestrus stage during the extinction phase were analized. This suggests that the higher fear responses observed during extinction is most likely due to the interaction of morphine with the low estrogen cycle stage during conditioning and not due to the cycle stage during extinction. The fact that a single dose of morphine produced a transient over expression of fear memories in metaestrus when administered immediately after conditioning but not before extinction, supports that morphine’s effect is related to memory processes that occur immediately after the trauma. This suggests that ovarian hormones and morphine interact to alter fear memory, when morphine is given close to the trauma event. However, once morphine is no longer present, animals recover and are able to show fear responses comparable to the animals that did not receive morphine.

Animal studies have examined the effects of acute morphine on male rats, on various fear conditioning behavioral protocols. Glover and Davis (2008) study demonstrated that morphine facilitates extinction in male rats exposed to fear potentiated startle. Although one of the morphine doses they used is the one we used in our study (2.5 mg/kg), the timing of s.c injection of morphine and the differences in the behavioral protocol do not allow us to fully compare their findings to our study. Szczytkowski-Thomson et al. (2013), on the other hand used a single higher dose of morphine (15 mg/kg), which was administered immediately after a stressor to males, but it did not reduce the fear response. In the same study, repeated morphine doses (7.5 mg/kg) cause a decrease in fear (Szczytkowski-Thomson et al., 2013). We decided not to treat repeatedly with morphine due to dependence and withdrawal issues, as we wanted to maintain a clinically relevant study. Our current model was designed considering the translational potential of morphine as a treatment to lower the possibilities of trauma-related symptoms manifestation, but without creating dependence to the drug. Furthermore, there is no electric shock after the morphine administration in our protocol and this eliminates the possibility of morphine altering pain perception. In addition, post-trial morphine administration using the passive avoidance paradigm, has been shown to produce facilitation of fear memories (Mondadori and Waser, 1979). This study used very high doses of morphine (40 or 100 mg/kg) which may produce withdrawal signs in animals. Unfoltunately, they did not test females. However, we acknowledge the possibility of a post-trial reinforcement effect (Mondadori et al., 1977; Huston and Mueller, 1978), but we are inclined to think that it is related to a morphine-estrous cycle interaction, since the same behavioral outcome was not observed in males or proestrus cycling females. This possibility requires further comparative studies.

Western blots revealed that in response to morphine and extinction, males and proestrus females showed an increase in the expression of MOR in the amygdala, but this was not observed in metaestrus females. This parallels our behavioral findings showing that males and proestrus females had no differences in freezing responses compared to saline controls, but metaestrus females showed over expression of freezing. As a site of initial acquisition of extinction, it might be expected that the BLA is also a site of extinction consolidation. Intercalated (ITC) amygdala neurons constitute the likely mediators of extinction because they receive conditioned stimulus information from the BLA and contribute inhibitory projections to the central nucleus (CEA), the main output station of the amygdala for conditioned fear responses (Likhtik et al., 2008). The majority of MORs are localized in the ITCs (Likhtik et al., 2008). Therefore, we propose that an increase in MOR activity within the ITC might decrease fear response levels to that of saline animals, as observed in this study, but the presence of high ovarian hormones in females and testosterone in males is necessary for this increase to occur. Taken together, these results suggest that MORs plays a role in the molecular events underlying fear extinction. However, given the fact that proestrus and metaestrus female rats differ in their ovarian hormone levels, it is possible that the observed behavioral responses are also influenced by these hormonal differences.

In addition to the amygdala, opioids are released in the ventrolateral PAG when the animals are exhibiting fear to the conditioned stimulus during the early phases of extinction (Parsons et al., 2010). This suggests that the opioid system plays an important role in fear conditioning and extinction. However, we did not see significant results in the expression of MOR when rats were sacrificed immediately after extinction. Two possible explanations for our results are that by the time we sacrificed the animals (after extinction) those changes have already occurred; or the fact that we dissected the whole PAG instead of ventrolateral region alone thus masking what is happening in the ventrolateral PAG *per se*.

In conclusion, our data suggest that females exposed to trauma during low ovarian hormone stages could be more vulnerable to over-expression of the traumatic memories. There are no data in the literature regarding which brain structures are directly involved in the estrogen-morphine interaction that modulate fear. However, our findings are beginning to fill this gap by providing data in two brain structures that are known to have an important role in fear conditioning, the PAG and the amygdala. We suggest that future studies should address in depth the interactions of ovarian hormones and opioid receptors activity that may lead to either protect or exacerbate trauma memories. This study contributes to clarifying the physiological role of morphine in memory consolidation as one of the first providing a description at molecular level in females and highlighting the protagonic role of the amygdala.

## Conflict of Interest Statement

The authors declare that the research was conducted in the absence of any commercial or financial relationships that could be construed as a potential conflict of interest.
